# Supplementary material for: STAT5-dependent regulation of CDC25A by miR-16 controls proliferation and differentiation in FLT3-ITD acute myeloid leukemia
Source: Sci Rep. 2020 Feb 5;10:1906. doi: 10.1038/s41598-020-58651-x (PMC7002454; doi:10.1038/s41598-020-58651-x)
Supplement: Supplementary file 1 — Supplementary information. [file 41598_2020_58651_MOESM1_ESM.pdf]

Supplementary informations for :

STAT5-dependent regulation of CDC25A by miR-16 controls proliferation and differentiation in FLT3-ITD acute myeloid leukemia

by

Gabrielle Sueur, Alison Boutet, Mathilde Gotanègre, Véronique Mansat- De Mas, Arnaud Besson, Stéphane Manenti and Sarah Bertoli.

Supplementary figure 1

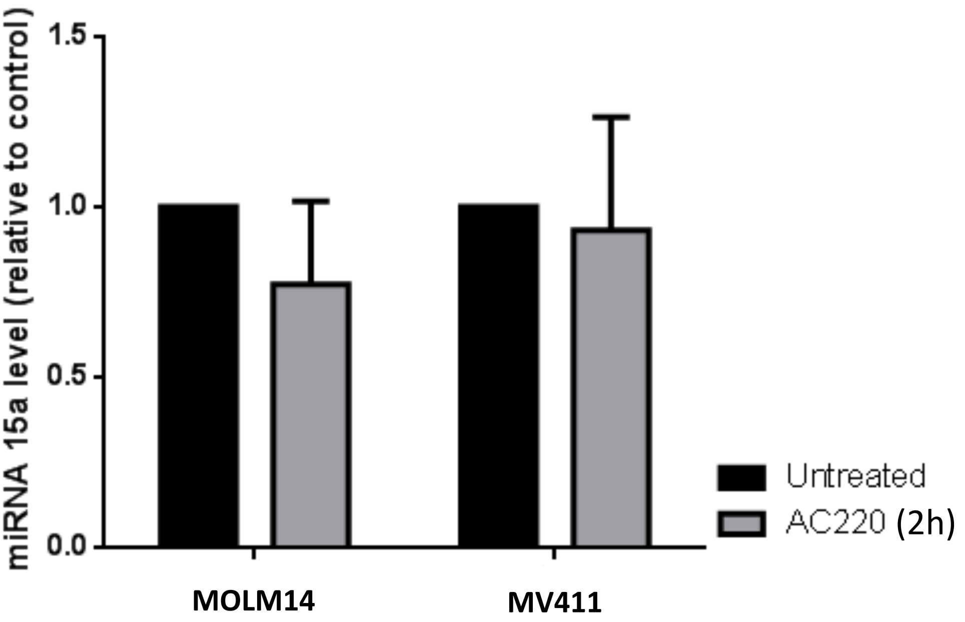

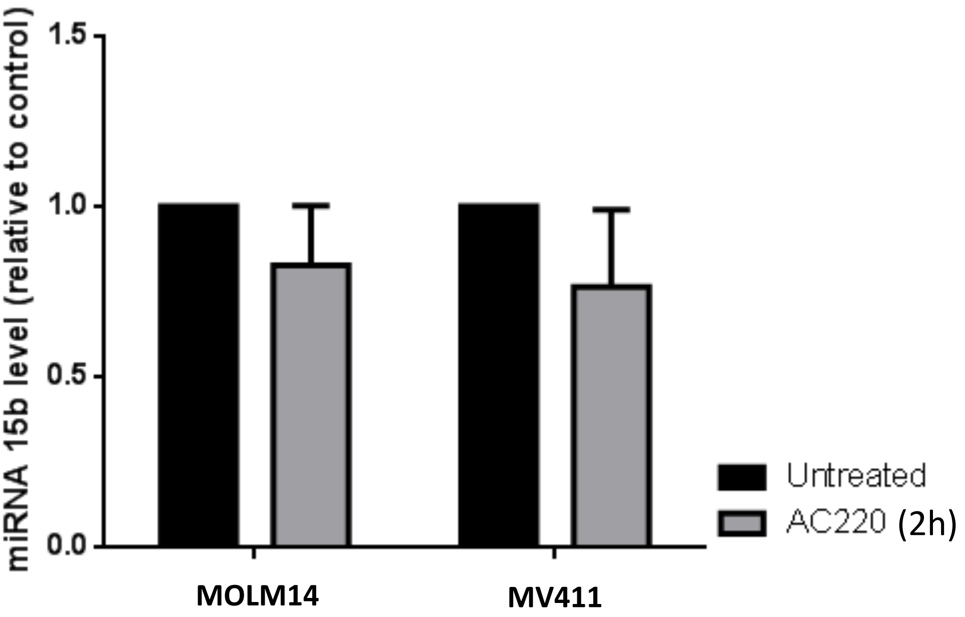

Supplementary figure 2

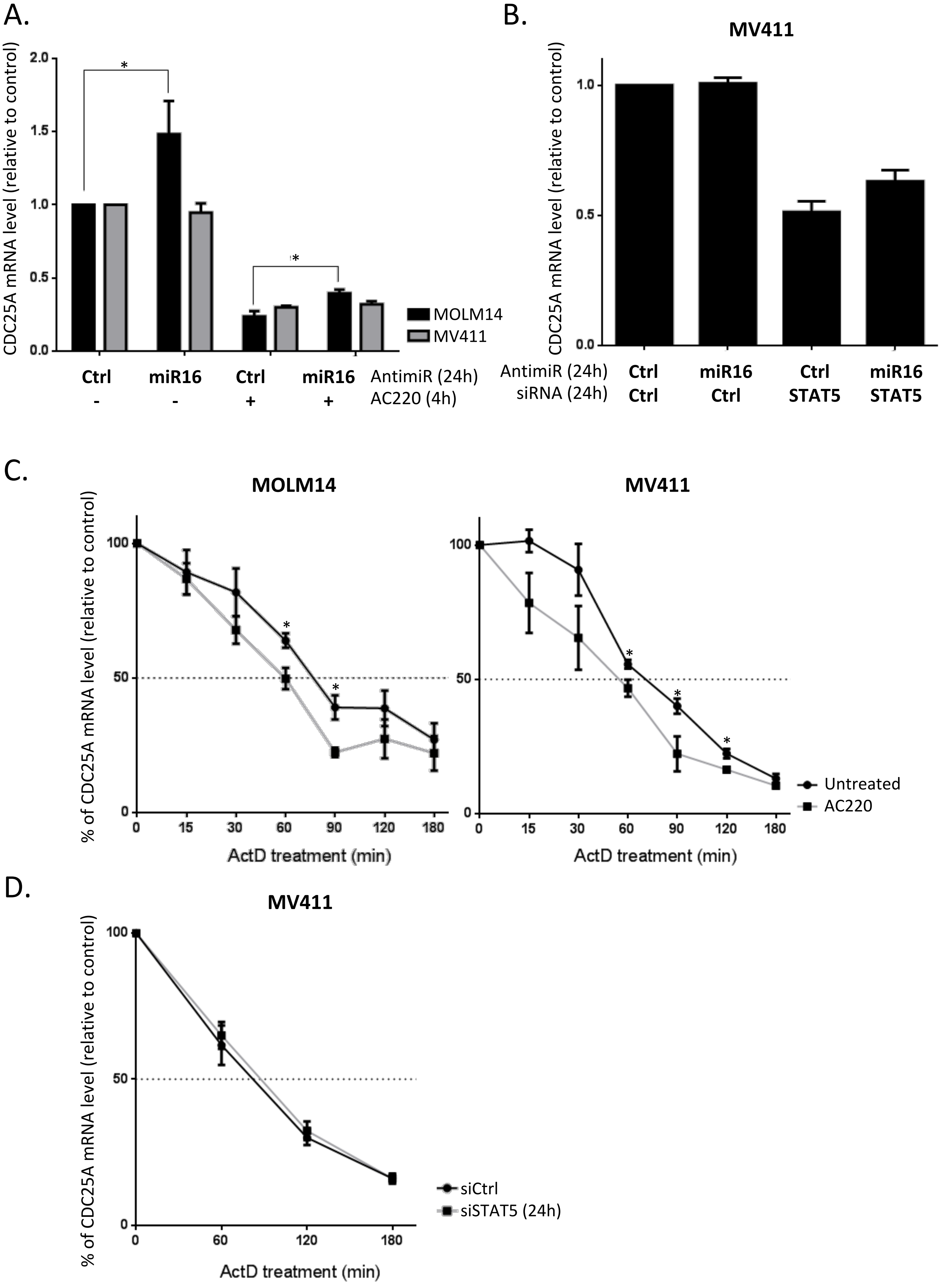

Supplementary Figure 3

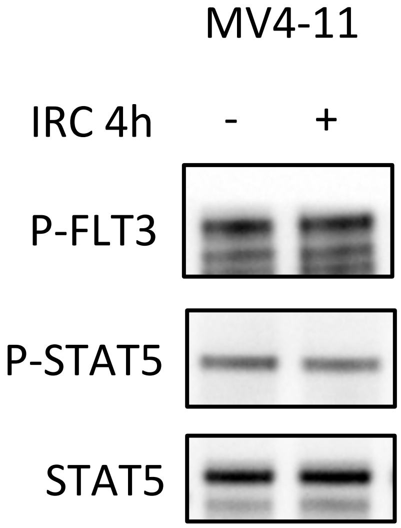

Supplementary figure 4

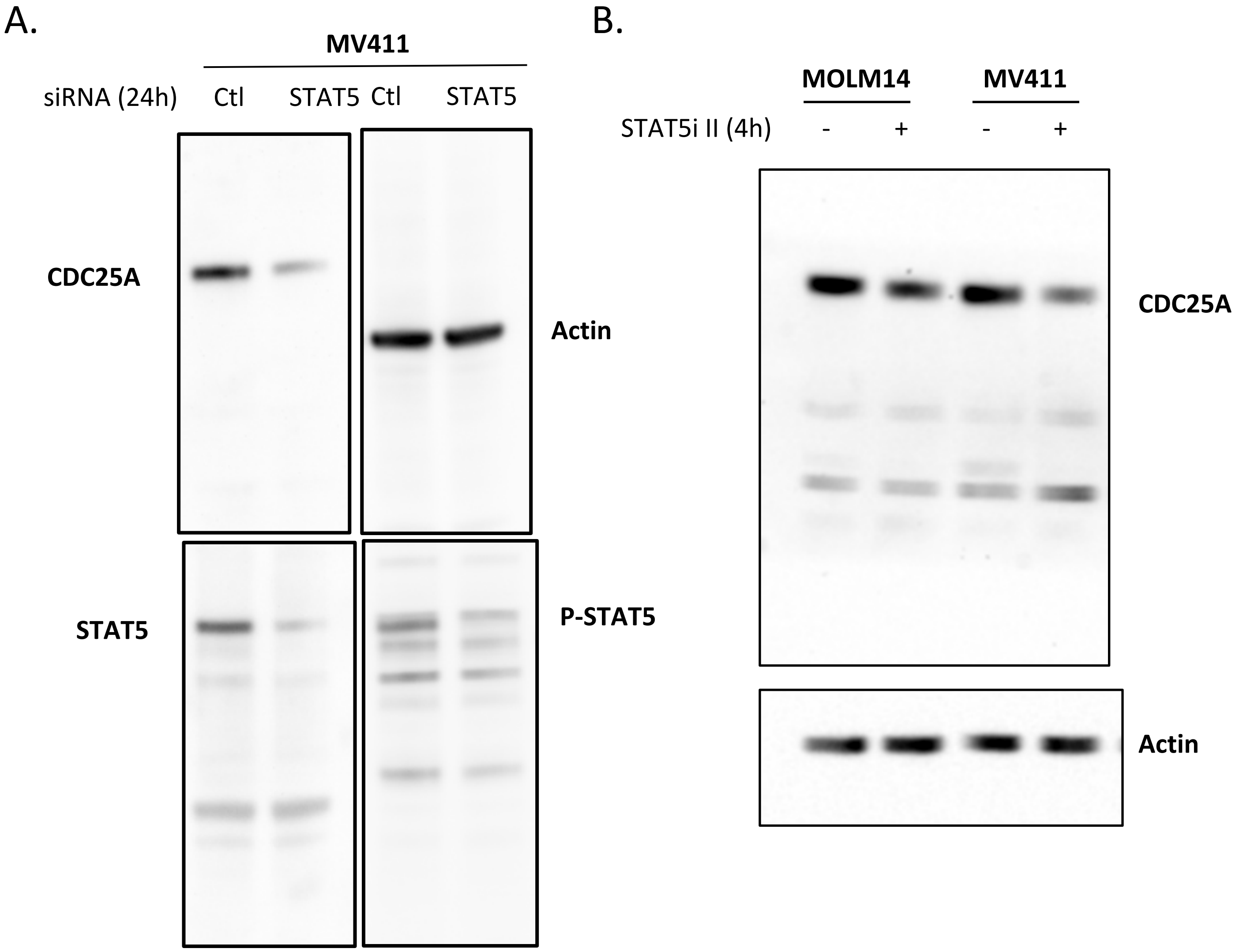

Supplementary figure 5

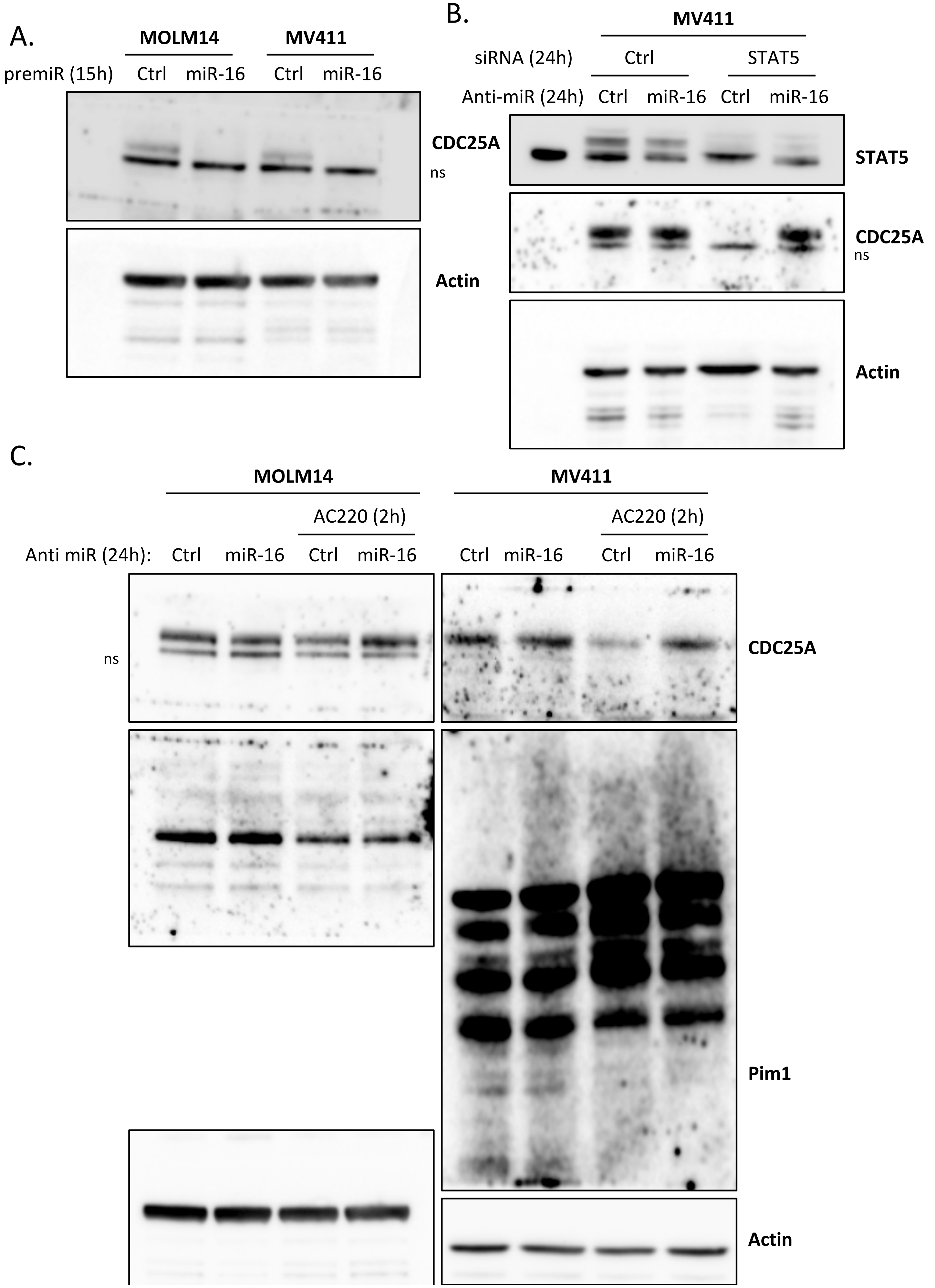

Supplementary figure 6

A.

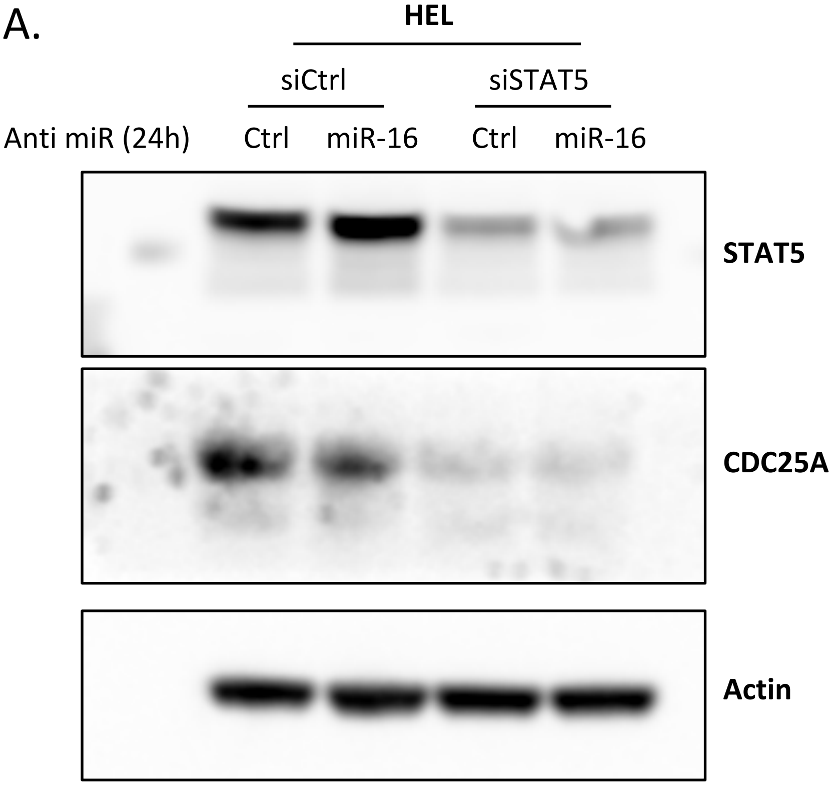

B.

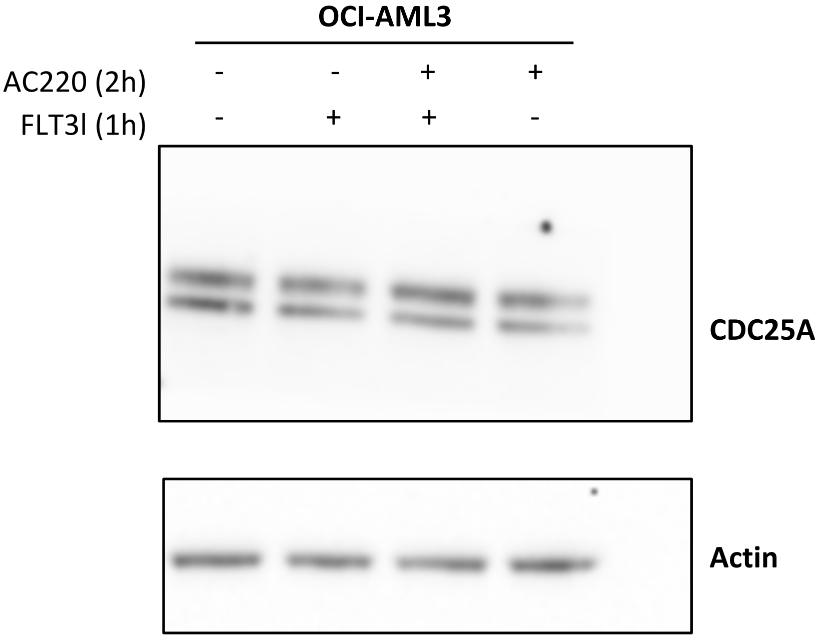

<u>Supplementary figures legends</u>

<u>Supplementary figure 1:</u> <u>FLT3 inhibition does not modulate miR-15-a or b level</u>

MOLM14 and MV411 cells were treated for 2h with AC220 (2nM) and miR-15-a (upper panel) and miR-15-b (lower panel) levels were analyzed by RT-qPCR. These results are representative of at least 3 independent experiments. Error bars represent the SEM.

<u>Supplementary figure 2</u>: <u>miR-16 tenuously regulates CDC25A mRNA level</u>

A. MOLM14 (black bars) and MV411 (grey bars) cells were transfected for 24 hours with a miR-16 inhibitor and treated for 2h with AC220 (2nM). The CDC25A mRNA level was analyzed by RT-qPCR

B. MV411 cells were transfected for 24 hours with a miR-16 inhibitor and a STAT5A/B siRNA. The CDC25A mRNA level was analyzed by RT-qPCR

C. MOLM14 and MV411 cells were treated for 1h with AC220 (2 nM) and further treated with D actinomycin (3µg/ml) for the indicated times. The CDC25A mRNA level was analyzed by RT-qPCR.

D. MV411 cells were transfected for 24h with STAT5A/B siRNA and treated with D actinomycin (3µg/ml) for the indicated times. The CDC25A mRNA level was analyzed by RT-qPCR.

These results are representative of at least 3 independent experiments. Error bars represent the SEM.

<u>Supplementary figure 3:</u> <u>CDC25A inhibition does not affect FLT3-ITD activity</u>

MV4-11 cells were treated for 4 hours with the CDC25 inhibitor IRC (200 nM), and the activity of the FLT3-ITD receptor was followed by western blot analysis of FLT3-ITD

phosphorylation (P-FLT3) and STAT5 phosphorylation (P-STAT5). Western blot analysis of STAT5 protein level was also performed (STAT5).

Supplementary figure 4: Full length blots for Figure 1

A. Full length blots for Figure 1.A

B. Full length blots for Figure 1.B

Supplementary figure 5: Full length blots for Figure 3

A. Full length blots for Figure 3.E

B. Full length blots for Figure 3.F

C. Full length blots for Figure 3.G

Supplementary figure 6: Full length blots for Figure 4

A. Full length blots for Figure 4.A

B. Full length blots for Figure 4.C

	Forward
CDC25A TATA Box 21/105	CCGCTATTACCGCGAAAGG
STAT site in the CDC25A promoter -186/59	CTTCTGAGAGCCGATGAC

Supplementary Table 1: Primers used in ChIP experiments (Fig 2.A)

Reverse
AGCTCCGGGTAGCAGAAA
CAAACGGAATCCACCAATC

Sample	FLT3 status	Karyotype abnormalities
WT #1	WT	NA
WT #2	WT	NA
WT #3	WT	NA
WT #4	WT	None
WT #5	WT	None
WT #6	WT	None
WT #7	WT	None
WT #8	WT	NA
WT #9	WT	None
WT #10	WT	None
WT #11	WT	None
WT #12	WT	None
ITD #1	ITD 62%	46,XY,add(14)(q3?1)[6]/46,XY[14]
ITD #2	ITD 64.7%	47,XX,+8[22]
ITD #3	ITD 30%	NA
ITD #4	ITD 101%	None
ITD #5	ITD 70%	NA
ITD #6	ITD 6%	NA
ITD #7	ITD 59%	NA
ITD #8	ITD 51%	NA
ITD #9	ITD 45%	46,XY,del(20)(q11)<22>
ITD #10	ITD 20%	None
ITD #11	ITD 55%	None
ITD #12	ITD 20%	None
ITD #13	ITD 81%	None
ITD #14	ITD 105%	None
ITD #15	ITD 80%	None
ITD #16	ITD 68%	None

Supplementary Table 2: Summary of the known characteristics for AML patient samples used in Fig. 3B and 5C.
NA: Not Available, WT: Wild-Type
